# Immunogenicity of Fetal Liver-Derived Progenitor and Stem Cells: Expression of MHC Molecules 

**DOI:** 10.31557/APJCP.2026.27.1.297

**Published:** 2026-01-21

**Authors:** Denys Vatlitsov, Khrystyna Sorochynska, Alla Tkachenko, Nadiia Dovhopola, Mariya Klunnyk

**Affiliations:** 1 *State Institution “National Research Center for Radiation Medicine, Hematology and Oncology of National Academy of Medical Sciences of Ukraine”; Laboratory of Molecular Biology of the Department of Clinical Immunology of the Institute of Clinical Radiology; Research and Development Laboratory, Limited Liability Company “Cell Therapy Center “EmCell”, Kyiv, Ukraine.*; 2 *Stem Cell Bank CEO, Laboratory Department, Limited Liability Company “Cell Therapy Center “EmCell”, Kyiv, Ukraine.*; 3 *Biotechnology Laboratory, Limited Liability Company “Cell Therapy Center “EmCell”, Kyiv, Ukraine. *; 4 *Research and Development Laboratory, Limited Liability Company “Cell Therapy Center “EmCell”, Kyiv, Ukraine.*; 5 *Clinical Department, Limited Liability Company “Embryonic Tissues Center “EmCell”, Kyiv, Ukraine.*

**Keywords:** Immunogenicity, Fetal Liver Cells, HLA Expression, Stem Cell Therapy, Regenerative Medicine

## Abstract

**Background:**

Regenerative medicine increasingly relies on stem cell–based therapies, yet their clinical success is largely determined by immunological compatibility. Fetal liver–derived progenitor and stem cells represent a promising, yet insufficiently characterized, source for transplantation.

**Objective:**

This study aimed to evaluate the immunogenicity of fetal liver cells by analyzing the expression of *HLA* class I and II molecules and detecting the presence of anti-*HLA* antibodies after cryopreservation at early gestational ages.

**Methods:**

Cell suspensions were obtained from fetal livers at 5–12 weeks of gestation. Flow cytometry was performed using a CyFlow Space cytometer (Sysmex, Germany), and results were analyzed with Statistica 10. *HLA* class I (*HLA*-ABC) and class II (*HLA*-DR/DP/DQ) expression was quantified as the percentage of positive cells and their mean fluorescence intensity. Anti-*HLA* antibodies were assessed in the cell suspensions. Statistical analysis included descriptive statistics, group comparisons, and correlation analyses with gestational age.

**Results:**

*HLA* class I expression was consistently detectable across all samples. In contrast, *HLA* class II expression increased progressively with gestational age, reflecting the developmental maturation of the fetal immune system. Importantly, these antigens primarily indicated differentiation toward myeloid lineages, which are unlikely to provoke graft-versus-host immune reactions. Anti-*HLA* antibodies were not detected in any of the analyzed suspensions.

**Conclusions:**

This study provides the first systematic assessment of *HLA* expression in fetal liver–derived progenitor and stem cells following cryopreservation. The results suggest that these cells retain a favorable immunological profile, supporting their potential application in regenerative medicine. The findings highlight the importance of considering gestational age in evaluating immunogenicity. Further studies with larger sample sizes and in vivo validation are required to confirm clinical safety.

## Introduction

Regenerative medicine represents one of the most promising fields of modern biomedical research, offering therapeutic opportunities for patients with severe or otherwise untreatable conditions. Among the available strategies, stem cell–based therapies hold particular potential due to their ability to modulate inflammation, promote angiogenesis, and differentiate into multiple cell lineages [1]. However, the successful translation of stem cell therapies into clinical practice is largely dependent on their immunological safety.

The immune response to transplanted cells is primarily determined by the expression of human leukocyte antigens (*HLA*) [2, 3, 4]. While pluripotent stem cells have been widely studied, their application remains limited by the risk of immune rejection mediated by *HLA* class I and II molecules [5, 6, 7, 8]. Recent reviews emphasize that even in advanced stem cell platforms, *HLA* incompatibility continues to pose a critical barrier to clinical translation [9]. Moreover, organoid and tissue-engineering studies demonstrate that both *HLA* class I and II antigens can trigger strong T- and B-cell responses, raising concerns regarding long-term graft tolerance [10].

In parallel, innovative strategies are being developed to reduce the immunogenicity of stem cells, including genetic deletion of *HLA* molecules and the expression of modified *HLA*-G isoforms [11, 12]. These approaches underscore the growing scientific consensus that minimizing immunogenicity is essential for the safe and effective use of stem cell therapies. At the same time, certain cell types, such as embryonic stem cell-derived keratinocytes, have been shown to exhibit inherently low immunogenicity [13].

Despite this progress, there is still limited knowledge about the immunological characteristics of fetal liver–derived progenitor and stem cells. These cells, which represent an early stage of hematopoietic and hepatic development, may possess unique immunological properties distinct from other stem cell sources. Assessing their *HLA* expression patterns and potential for eliciting immune responses is therefore critical for determining their safety in clinical applications.

The novelty of the present study lies in providing original data on *HLA* class I and II expression in fetal liver cells at early gestational ages (5–12 weeks) after cryogenic storage. To our knowledge, such analysis has not been systematically conducted before. By addressing this gap, our research contributes to the broader understanding of fetal stem cell immunogenicity and offers evidence to guide the development of safer cell-based therapies.

### Review of Recent Studies

Recent advances in regenerative medicine have emphasized the importance of immunological safety in stem cell–based therapies. One of the central challenges is the expression of human leukocyte antigens (*HLA*), which determines the extent of immune recognition and rejection of transplanted cells.

A number of recent studies have highlighted the dual role of *HLA* molecules in stem cell transplantation. A comprehensive review by Yoshihara and colleagues (2021) underscored that *HLA*-mediated immune responses remain one of the key barriers to clinical application of pluripotent stem cells, despite progress in modulating host tolerance [9]. In line with this, organoid transplantation models demonstrated that both *HLA* class I and class II antigens trigger robust T- and B-cell responses, suggesting the need for strategies to minimize alloimmunization risk (Xu et al., 2025) [10].

One of the promising approaches involves genetic engineering of stem cells to decrease their immunogenicity. Recent work on hypoimmunogenic induced pluripotent stem cells (iPSCs) employed CRISPR/Cas9-mediated deletion of *HLA* class I and class II molecules, achieving reduced recognition by the host immune system (Kim et al., 2025) [11]. Complementary findings indicate that the expression of mutated forms of *HLA*-G may further contribute to immune evasion in human embryonic stem cells, offering a potential strategy for enhancing transplant safety (Zhang et al., 2025) [12].

At the same time, some evidence suggests that not all stem cell types are equally immunogenic. For instance, keratinocytes derived from human embryonic stem cells displayed low *HLA* expression and limited activation of allogeneic immune responses, confirming that cellular phenotype and differentiation stage critically shape immunological outcomes (Liu et al., 2024) [13].

Previous studies examining fetal stem cells’ ability to provoke immune responses in recipients typically used test material obtained during the second and third trimesters [1, 14, 15, 16–18].

It is known that during the first trimester of pregnancy, fetal cells exhibit very low *HLA* molecule expression [6, 19, 20]. Furthermore, placental tissues express a non-classical pattern of *HLA* class I molecules and do not express *HLA* class II molecules, which is considered a key mechanism for fetal immune evasion from maternal recognition [7, 21, 22]. Recently, the potential presence of recipient-specific antibodies to *HLA* (RSA) in donor material has also been raised as a concern.

Taken together, these findings provide a broader context for evaluating the immunogenicity of fetal liver–derived progenitor and stem cells. They highlight both the risks associated with *HLA* expression and the emerging strategies to mitigate immune rejection, underscoring the importance of assessing immunological safety in early-stage therapeutic development.

The aim of the present study was to assess the immunologic safety of fetal stem cell-based therapy. This involved evaluating *HLA* class I and II expression on fetal liver cells and the presence of anti-*HLA* antibodies in suspensions of fetal liver cells from 5 to 12 weeks of gestation following cryogenic storage.

## Materials and Methods

### Study design and overview

This laboratory study evaluated the immunological safety of fetal liver–derived progenitor and stem cells by quantifying *HLA* class I (*HLA*-ABC) and *HLA* class II (*HLA*-DR/DP/DQ) expression and screening for anti-*HLA* antibodies in cell suspensions after cryogenic storage. Gestational ages ranged from 5 to 12 weeks. The primary endpoint was the proportion of cells expressing *HLA* class I/II and the corresponding mean fluorescence intensity (MFI); a secondary endpoint was the presence/absence of anti-*HLA* antibodies in cell suspensions.

### Material source and eligibility

#### The cells source

Liver cell suspension samples were obtained from fetuses at 5 to 12 weeks of gestation. These samples were sourced from medical facilities following legally performed elective abortions in healthy women, conducted for social reasons. All female donors underwent preliminary screening for blood-borne diseases. Tissue donations complied with the ethical and legal standards of Ukraine and adhered to the World Medical Association Declaration of Helsinki Ethical Principles for Medical Research Involving Human Subjects [23]. Participation of healthy volunteers in the study was conducted in accordance with national standards and protocols [23, 24]. Peripheral blood samples from 16 healthy volunteers, aged 34.5 ± 12.6 years (range: 26-64 years), were used as control samples.

### Sampling

Cell suspensions were sampled from a cryobank, where they had been stored in liquid nitrogen at -196°C. Preparation of these cell suspensions followed the specific guidelines of a patented protocol [25]. Samples were randomly selected from each gestational week, ensuring compliance with conditions of storage duration, microbiological sterility, viral negativity, and cell viability exceeding 90%. For each gestational week, three 1 mL samples were used, each with a concentration of 8.5 ± 3.5 × 106 cells/mL. All cell suspensions were confirmed to be free of bacterial, fungal, and viral pathogens, including a wide range of concerns such as HIV-1, HIV-2, HPV, HBV, HCV, EBV, CMV, HHV6, HSV-1/2, Treponema pallidum, Rubella virus, Parvovirus B19, Mycoplasma genitalium, Toxoplasma gondii, Chlamydia trachomatis, Ureaplasma parvum, and Ureaplasma urealyticum. The period of cryopreservation did not exceed three months.

### Flow cytometry

Flow cytometric analysis was performed on a CyFlow Space cytometer (Sysmex, Germany) equipped with a 488 nm laser. Cell suspensions were stained with PE-labeled monoclonal antibodies against *HLA* class I (*HLA*-A, -B, -C; clone EMR8-5, Cat. no. 567581, BD Pharmingen, USA) and FITC-labeled antibodies against *HLA* class II (*HLA*-DR, -DP, -DQ; clone TU39, Cat. no. 555558, BD Pharmingen, USA). Propidium iodide (PI; Cat. no. 537060, Calbiochem, USA) was used to assess cell viability.

Anti-*HLA* class I and II antibodies were assessed using Flow PRA test kits (class I, Cat. no. FL1SP; class II, Cat. no. FL2SP; One Lambda Inc., USA), which are FDA-approved for in vitro diagnostics, following the manufacturer’s instructions.

Data acquisition was conducted using FlowMax 2.9 software (Quantum Analysis GmbH, Germany), and data analysis was performed in Statistica 10 (StatSoft, USA).

### MHC Class I and Class II antigen detection by flow cytometry

MHC antigen expression on fetal liver cells was evaluated using flow cytometry with specific anti-*HLA* antibodies. PE-labeled monoclonal antibodies targeted Class I *HLA* (A, B, and C) (clone EMR8-5), while FITC-labeled antibodies targeted Class II *HLA* (DR, DP, and DQ) antigens (clone TU39) (BD Pharmingen, USA). *HLA*-DM and *HLA*-DO were excluded due to their intracellular localization [2, 4, 26]. The FITC Mouse IgG2a Isotype Control (BD Pharmingen, USA) was used as an isotype control, and peripheral blood cells from healthy donors served as controls. Erythrocytes were lysed using Lysing Solution 10X Concentrate CE/IVD (BD Pharmingen, USA).

### Anti-HLA I and HLA II antibody detection by flow cytometry

The assessment of anti-*HLA* Class I and II antibodies was conducted using Flow PRA test kits from One Lambda Inc. (USA), approved by the FDA for in vitro diagnostics. These kits feature beads coated with 32 specific *HLA* antigens per class, divided into four groups, each coated with eight antigen types, including control beads without antigens. Each sample was analyzed against all antigen groups for both *HLA* Class I and II. The binding of sample antibodies to the beads was detected using FITC-conjugated anti-human IgG. A positive reaction was indicated by a shift in the FL1 channel relative to the negative control, and antigen specificity was detected via the FL2 channel. The evaluation was performed based on the kit instructions [27], with 5 µL of beads from each group mixed with 20 µL of the cell suspension supernatant. Samples were incubated for 30 minutes at room temperature in the dark, followed by protocol-guided washing. FITC-conjugated anti-human IgG was added and incubated for another 30 minutes in the dark. Finally, cells were fixed with 0.5 mL of fixing solution. Bead populations were gated on an FSC vs. SSC dot plot, with at least 5000 gated events acquired per sample.

These carefully controlled procedures ensure accurate assessments in studying *HLA* presentations and antibody interactions, vital for insights into the immunological compatibility of fetal cell therapies. Green (FL1) and orange (FL2) fluorescence intensities were assessed for the gated beads. Both negative and positive controls were utilized, and fluorescence compensation was adjusted for each experimental run. Untreated beads from each group were used to establish the cut-off threshold on the FL1 channel. A gating strategy was established for each group of untreated beads to facilitate further result analysis. Positive sample reactions were categorized into several levels of reactivity: strong, intermediate, and weak. The kit also allows for results to be classified as “negative” or “weakly positive”.

### Statistical analysis

Descriptive statistics were calculated as mean ± standard deviation (M ± SD). The proportion of cells expressing *HLA*-ABC and *HLA*-DR/DP/DQ antigens was determined for each gestational age group (5–12 weeks). Results are presented in Table 1, which shows the distribution of positive and negative phenotypes across gestational weeks.

Comparisons across gestational age groups were performed using ANOVA/Kruskal–Wallis test with post-hoc analysis, depending on data distribution. Associations between gestational age and *HLA* expression were evaluated by Spearman’s rank correlation. A two-sided p < 0.05 was considered statistically significant. Data were analyzed using Statistica 10 (StatSoft, USA).

## Results

### The fetal liver cells do not present MHC Class I HLA-ABC and present a negligible amount of MHC Class II HLA-DR, DP and most DQ antigens

The antigen profile of fetal liver cells from 5 to 12 weeks of gestation was investigated using monoclonal antibodies specific for MHC Class I (*HLA*-ABC) and Class II (*HLA*-DR, DP, DQ) antigens to perform *HLA* phenotyping. For MHC Class I *HLA*-ABC, antibodies showed negative or weakly positive signals, with interaction affecting less than 0.5% of fetal liver cells on average across all gestational ages studied (mean ± standard deviation). Regarding MHC Class II antigens, the average percentage of *HLA*-DR, DP, and most DQ positive fetal liver cells increased from 0.91 ± 0.32% at week 5 to 3.68 ± 0.92% at week 12 (see Table 1). This trend aligns with findings by Edwards et al. (1985, 1986), who observed similar patterns in fetal spleen cells.

To assess the significance of these antigen expression levels, *HLA* expression was compared to that in donor peripheral blood cells, using the positive signal level as a reference point. This comparison helps establish the relative antigen presentation capabilities of fetal liver cells.

Peripheral blood cell samples exhibited significantly higher fluorescence signal levels for MHC Class II antigens *HLA*-DR, DP, and most DQ, registering at 139.03±5.33 units, compared to fetal liver cells which measured at 114.86±3.38 units, with a negative control baseline of 32.55±2.36 units. The fluorescent signal intensity is proportional to the amount of fluorophore-labeled antibodies bound to antigens, indicating a substantially lower *HLA* expression in fetal liver cells when analyzed under identical flow cytometry parameters. This suggests that differences in antigen presentation are due to cellular properties other than size or surface area.

### The fetal liver cell suspensions do not demonstrate anti-HLA Class I and anti-HLA Class II antibody reactivity

The anti-*HLA* antibody investigation was conducted in two phases. First, cell suspensions stored in a cryobank for a minimum of three months post-processing were analyzed to ensure comprehensive evaluation over the maximal storage period required to verify parameters and ensure safety. In the second phase, samples obtained directly during cellular preparation, without cryopreservation, were evaluated. Results were documented based on positive bead shifts, and percentage values for positive beads were calculated for each gestational week. Conclusions regarding the presence of each of the 64 anti-*HLA* antibodies in the sample dispersing medium were drawn. An overall mean value of positive bead shifts was calculated for each group of both *HLA* classes and all antigens, and an antigen reaction map was generated for each sample.

Despite slight signal shifts observed through antibody typing of fetal liver cell suspension samples, these values were classified as “negative” according to the kit manual, occasionally nearing the “weak positive” cutoff in rare instances. No significant differences were noted between native and cryopreserved samples regarding anti-*HLA* Class I and II antibody levels, thus collective mean values are provided.

According to the reactivity evaluation table, the observed values matched the “negative or weak positive” category, affirming their classification as negative. For anti-*HLA* Class I antibodies, the highest shifts were noted in samples from the 9th, 10th, and 11th gestational weeks. Specifically, a signal shift was observed in 12.60 ± 4.24% of beads coated with A11:01, A29:02, B18:01, B38:01, BW6, BW4, C12:03 antigens for the 9th week of gestation (group 1, beads #2); 12.89 ± 4.35% and 12.59 ± 2.1% in beads coated with A24:02/50/54/55/56/58, A30:02, B37:01, B48:01, BW4, BW6, C06:02, C08:01 (group 4, beads #1), and A03:01/14/20, A32:01, B27:05, B40:01/55, BW4, BW6, C02:02/29, C03:04 (group 3, beads #1) respectively for the 10th week; and 12.04 ± 3.68% for beads coated with A03:01, A66:01, B15:03, B52:01, BW6, BW4, C02:10, C12:02 for the 11th week (see Supplementary Table 1).

Among anti-*HLA* Class II antibody estimations, the highest reactivity level of 10.88 ± 2.59% was seen in beads coated with DRB101:01, DRB116:01, DQB105:01, DQB105:02, DQA101:01, DQA101:02, DPB103:01, DPB1*04:01 antigens (group 4, beads #6) (see Table 2).

## Discussion

Fetal stem cells offer a promising source for regenerative medicine due to their genetic stability – carrying fewer mutations – and less extensive epigenetic modification compared to adult cells. These attributes make them highly suitable for expanding in culture, overcoming the ex vivo expansion limitations faced by adult cells [8, 28, 29, 30]. The fetal liver, in particular, is a rich source of hepatic, mesenchymal, and hematopoietic stem cells, which hold significant potential for stem cell-based therapies [2, 16, 31]. However, the immunogenic profile of fetal liver stem cells and their potential to elicit an immune response in recipients remain partially understood.

*HLA* molecules play a central role in organ and tissue transplantation by distinguishing self from non-self-proteins, making donor-recipient histocompatibility a crucial aspect of allotransplantation [32, 33, 34]. Ensuring compatibility involves stringent evaluation of grafts against recipient profiles to accurately assess histocompatibility [35, 36]. This study employed a two-stage design to evaluate the immunological safety of fetal liver cell samples from 5 to 12 weeks of gestation. The design was informed by the limited immune response of maternal organisms to fetal cells and the low expression levels of *HLA* antigens on fetal cells.

The fetal adaptive immune system avoids maternal rejection due to its functional immaturity, limited antigen exposure, and insufficient immunological memory. During development, the fetal immune system learns to tolerate benign or necessary antigens transferred across the placenta. Notably, hematopoietic cells of maternal origin frequently exist within fetal tissues, and maternal microchimerism can persist into adulthood [37].

For stem cell therapies, safety and efficacy are ensured by the absence of donor-recipient cell cross-reactivity or complementarity. To assess the immunological safety of fetal liver cell suspensions, we evaluated *HLA* Class I and II expressions. Fetal liver samples showed negative or weakly positive signals, significantly lower than those in peripheral blood cells. Fetal liver cells primarily binding anti-*HLA*-DR, DP, DQ antibodies indicate myeloid lineage differentiation, including B-lymphocyte precursors, despite low expression levels [38, 39].

The present study provides novel insights into the immunological profile of fetal liver–derived progenitor and stem cells, focusing on the expression of *HLA* class I and II antigens at early stages of gestation after cryogenic storage. Our findings demonstrate a progressive increase in the proportion of cells expressing MHC class II antigens with advancing gestational age, while *HLA* class I expression was consistently observed. These results indicate the gradual maturation of the fetal immune system and highlight the potential safety considerations of using fetal liver cells in transplantation and regenerative medicine.

Our observations align with previous reports showing that *HLA* expression is a major determinant of graft immunogenicity [2, 8, 29, 30, 40–43]. A comprehensive review on pluripotent stem cells emphasized that *HLA* incompatibility continues to be a critical barrier to clinical application, even when using advanced culture and differentiation protocols [9]. Similarly, transplantation models using organoid-derived grafts confirmed that both *HLA* class I and II antigens can elicit robust alloimmune responses [10]. These findings reinforce the need for careful evaluation of *HLA* profiles when considering fetal stem cells for therapeutic use.

At the same time, strategies to overcome immunogenicity are rapidly evolving. For instance, gene-editing approaches have enabled the generation of hypoimmunogenic iPSCs through deletion of *HLA* molecules [11], and the introduction of mutated *HLA*-G isoforms has been shown to reduce immune recognition of embryonic stem cell–derived products [12]. In contrast, certain cell types such as embryonic stem cell–derived keratinocytes exhibit inherently low *HLA* expression, suggesting that specific developmental stages or lineages may confer reduced immunogenicity [13]. Compared with these approaches, fetal liver–derived progenitor cells offer the advantage of natural developmental immaturity, which may limit their immunogenicity without genetic modification.

Importantly, our study highlights a potential balance between developmental maturity and immunological safety. While progressive *HLA* class II expression reflects normal hematopoietic differentiation into myeloid lineages, these cells are unlikely to provoke graft-versus-host reactions due to their immunological characteristics. This observation supports the view that fetal liver cells, particularly at early gestational stages, could represent a promising and relatively safe source for cell therapy applications.

Nevertheless, several limitations must be acknowledged. The study was restricted to in vitro analysis of a limited number of samples, without in vivo validation of immune responses. Additionally, the use of cryopreserved suspensions, while clinically relevant, may not fully replicate the immunological profile of freshly isolated cells. Future research should therefore include larger sample sizes, functional assays of immune activation, and preclinical models to confirm the safety of fetal liver–derived stem cell transplantation.

In summary, our results expand the current understanding of immunogenicity in fetal stem cells, demonstrating for the first time the gestational age–related dynamics of *HLA* expression in fetal liver–derived progenitor cells after cryogenic storage. By situating these findings within the broader context of stem cell immunology and emerging hypoimmunogenic strategies, this work contributes original evidence to guide the safe application of fetal stem cells in regenerative medicine.

In conclusions, this study provides original evidence on the immunogenicity of fetal liver–derived progenitor and stem cells. We demonstrated that *HLA* class II expression progressively increases with gestational age, while *HLA* class I expression remains consistently detectable. These findings indicate that developmental maturation of the fetal immune system influences the immunological profile of fetal liver cells, but the observed antigens largely reflect hematopoietic differentiation into myeloid lineages, which are unlikely to trigger graft-versus-host responses.

The novelty of this work lies in the systematic assessment of *HLA* class I and II expression in fetal liver cells after cryogenic storage, filling an existing gap in the literature. Our data contribute to the broader understanding of stem cell immunogenicity and may inform the safe application of fetal progenitor cells in regenerative medicine.

Several limitations should be acknowledged. The study was based on a relatively small sample size, limited to in vitro flow cytometry analysis, and did not include in vivo confirmation of immune responses. Future studies involving larger cohorts, functional assays, and preclinical transplantation models are warranted to validate the clinical safety of these cells.

**Table 1:** Percentages of MHC Class I *HLA*-ABC and MHC Class II *HLA*-DR, DP, DQ Positive Fetal Liver Cells for Fetal Liver Cell Suspension Samples of 5-12^th^ Week of Gestation

Cells phenotype	The percentage of cells depending on the week of gestation, (M±SD %)					
	5	7	9	10	11	12
HLA-ABC^Pos^/	0.3	0.22	0.43	0.34	0.30	0.44
HLA-DR;DP;DQ^Neg^	±0.11	±0.11	±0.03	±0.21	±0.19	±0.12
HLA-ABC^Pos^/	0.29	0.24	0.28	0.14	0.24	0.29
HLA-DR;DP;DQ^Pos^	±0.10	±0.17	±0.03	±0.09	±0.11	±0.08
HLA-ABC^Neg^/	0.91	1.10	2.90	2.28	3.15	3.68
HLA-DR;DP;DQ^Pos^	±0.32	±0.51	±0.51	±0.91	±1.41	±0.92

Source: Data, acquired from a CyFlow Space flow cytometer (488 nm laser), were analyzed using descriptive statistical methods within Statistica 10

**Table 2:** Anti-HLA Class II Antibody Levels in Fetal Liver Suspension Samples of 5-12^th ^Week of Gestation (M±SD)

Group	Bead No.	Serogical Typing						HLA class II beads % higher than non-stained gates								
								NC	5^th^ week	7^th^ week	9^th^ week	10^th^ week	11^th^ week	12^th^ week	PC	
		DRB1		DRB3/4/5		DQB1										
1	1	1	4	NA	NA	7	5	2.86	5.8	8.29	7.42	10.19	4.98	6.28	99.95	
								±0,75	±1,32	±2,08	±0,61	±1,84	±1,32	±1,63	±2,95	
	2	18	12	52	NA	4	5	2.71	6.09	3.5	9.3	4.94	5.11	4.8	99.91	
								±0,87	±0,49	±2,01	±0,48	±0,52	±1,04	±1,32	±2,21	
	3	9	10	53	NA	9	5	1.02	3.66	7.62	9.59	10.48	8.4	4.38	99.91	
								±0,51	±1,57	±1,57	±2,33	±0,7	±2,03	±2,18	±1,95	
	4	103	7	NA	53	2	5	0.91	5.08	4.89	6.78	9.07	5.57	8.92	99.95	
								±0,3	±1,26	±1,6	±1,43	±2,02	±0,69	±2,74	±2,95	
	5	17	8	52	NA	2	4	0.49	3.19	3.96	5.13	5.21	9.05	4.15	99.94	
								±0,24	±1,82	±2,42	±2,34	±0,53	±2,69	±0,75	±1,55	
	6	4	15	53	51	8	6	2.81	7.09	9.13	3.4	3.34	8.16	3.5	99.94	
								±0,29	±0,65	±0,93	±1,93	±2,65	±1,57	±0.99	±2,92	
	7	103	17	NA	52	2	5	0.85	10.41	6.1	8.08	7.54	4.84	4.1	99.94	
								±0,42	±1,71	±0,57	±0,65	±1,87	±1,58	±0,49	±1,58	
	8	NA	NA	NA	NA	NA	NA	0.62	0.38	0.18	0.08	1.03	0.29	0.27	0.1	
								±0,35	±0,18	±0,05	±0,07	±0,46	±0,05	±0,11	±0,08	
	9	9	NA	53	NA	9	NA	0.62	8.7	6.99	8.27	6.51	7.13	5.25	99.98	
								±0,5	±0,69	±0,48	±0,71	±2,83	±2,72	±0,83	±2,81	
2	1	4	8	53	NA	8	4	1.66	6.03	9.98	5.07	6.15	6.26	7.81	99.92	
								±0,38	±1,43	±2,31	±1,69	±0,84	±1,86	±1,97	±2,45	
	2	17	7	52	53	2	NA	2.67	8.89	4.02	7.52	6.33	6.04	4.91	99.91	
								±0,24	±1,21	±1,14	±0,8	±0,57	±2,03	±0,9	±0,45	
	3	1	8	NA	NA	4	5	1.49	10	9	10.22	10.32	3.55	4.25	99.98	
								±0,08	±0,47	±2,23	±2,08	±1,85	±1,1	±2,02	±2,13	
	4	11	12	52	NA	7	6	0.42	7.8	8.95	10.15	7.25	8.21	4.32	99.94	
								±0,81	±1,6	±1,66	±0,74	±0,5	±0,58	±2,01	±2,09	
	5	7	11	53	52	2	7	2.77	9.87	4.49	3.92	5.9	8.44	10.03	99.95	
								±0,05	±0,65	±1,77	±1,81	±1,36	±2,37	±2,53	±0,82	
	6	4	11	53	52	7	8	0.31	4.49	10.4	7.31	6.64	9.76	8.19	99.92	
								±0,89	±1,86	±1,65	±1,85	±0,88	±0,98	±1,89	±0,58	
								NC	5^th^ week	7^th^ week	9^th^ week	10^th^ week	11^th^ week	12^th^ week		PC	
2	7	17	13	52	NA	2	6	2.17	5.69	7.96	7.65	6.3	3.91	5.15		99.9	
								±0,19	±1,77	±1,67	±0,48	±1,32	±0,45	±0,62		±1,95	
	8	NA	NA	NA	NA	NA	NA	1.32	0.69	0.05	1.1	1.4	1.12	0.18		0.53	
								±0,88	±0,15	±0,05	±0,14	±0,72	±0,38	±0,03		±0,15	
	9	14	16	52	51	7	NA	1.42	10.19	4.87	3.14	6.47	10.54	4.18		99.98	
								±0,13	±1,96	±2,37	±1,76	±2,78	±1,64	±0,74		±1,5	
3	1	1	11	NA	52	5	6	0.34	5.41	5.14	9.73	5.38	5.19	8.7		99.94	
								±0,48	±1,84	±2,07	±1,82	±2,95	±1,99	±2,97		±2,42	
	2	17	9	52	53	2	9	1.35	4.84	4.83	7.9	7.64	10.35	8.49		99.92	
								±0,64	±1,81	±0,53	±1,54	±1,79	±0,84	±1,6		±1,23	
	3	8	12	NA	52	9	4	0.94	7.25	4.13	4.03	6.82	3.27	8.75		99.99	
								±0,7	±1,46	±2,38	±0,75	±2,27	±1,5	±2,55		±1,74	
	4	15	16	51	NA	5	6	2.99	8.21	8.03	10.45	9.86	4.19	10.29		99.98	
								±0,01	±1,44	±0,93	±0,65	±2,98	±1,91	±0,99		±1,94	
	5	7	12	NA	52	9	5	1.1	9.76	5.58	7.12	9.36	10.82	7.98		99.92	
								±0,98	±0,49	±1,18	±2,34	±2,47	±1,3	±1,32		±1,41	
	6	7	10	53	NA	2	5	0.33	7.17	8.6	10.39	10.33	4.87	6.24		99.98	
								±0,86	±0,53	±2,14	±0,54	±1,82	±2,31	±1,45		±0,98	
	7	8	14	NA	52	4	5	0.28	7.91	3.73	5.19	9.28	10.72	10.66		99.93	
								±0,01	±0,86	±2,33	±1,93	±0,97	±2,97	±1,76		±2,04	
	8	NA	NA	NA	NA	NA	NA	0.96	0.59	0.61	0.46	0.46	0.46	0.96		0.03	
								±0,22	±0,13	±0,27	±0,05	±0,06	±0,12	±0,43		±0,02	
	9	9	15	53	NA	2	5	0.93	3.05	3.28	9.49	10.27	6.65	3.41		99.98	
								±0,76	±1,02	±1,67	±1,59	±2,79	±2,8	±1,43		±2,73	
4	1	18	7	52	53	2	4	1.9	6.27	4.66	6.16	7.06	9.79	6.89		99.91	
								±0,85	±1,03	±1,75	±1,23	±2,08	±0,71	±0,74		±2,84	
	2	18	10	52	NA	4	5	1.15	8.86	10.03	5.1	7.76	5.38	4.94		99.96	
								±0,35	±1,17	±1,26	±1,55	±1,2	±0,55	±2,94		±1,43	
	3	13	NA	52	NA	6	NA	0.3	7.87	6.35	9.24	10.17	6.3	3.75		99.92	
								NC	5th week	7th week	9th week	10th week	11th week	12th week		PC	
4	4	13	14	52	NA	5	6	2.63	5.55	9.34	10.24	8.58	5.49	4.69		99.34	
								±0,61	±1,76	±1,25	±1,87	±2,18	±2,23	±2,23		±2,99	
	5	16	13	51	52	5	7	1.92	5.77	6.84	3.82	7.73	6.99	4.6		99.92	
								±0,18	±0,94	±1,77	±2,39	±2,59	±1,18	±2,63		±1,4	
	6	1	16	NA	NA	5	NA	2.34	8.22	8.87	5.33	9.73	4.75	10.88		99.96	
								±0,29	±1,55	±1,78	±1,5	±1,15	±0,66	±2,59		±2,9	
	7	4	14	53	52	7	8	2.88	10.28	6.86	7.94	7.3	9.8	6.19		99.99	
								±0,28	±0,73	±0,53	±0,67	±1,25	±2,55	±1,57		±0,73	
	8	NA	NA	NA	NA	NA	NA	2.1	0.27	0.83	0.08	0.44	0.99	0.93		1.15	
								±0,43	±0,18	±0,28	±0,02	±0,25	±0,08	±0,56		±0,75	
	9	13	15	52	51	6	NA	1.79	3.05	5.37	6.86	9.16	5.62	6.61		99.92	
								±0,28	±1,77	±0,73	±1,46	±2,84	±0,7	±0,77		±1,8	
															

Source: Data, acquired from a CyFlow Space flow cytometer (488 nm laser), were analyzed using descriptive statistical methods within Statistica 10; Note: NC, negative control; PC, positive control; NA, not available

## Author Contribution Statement

Khrystyna Sorochynska: Conceptualization, Investigation, Methodology, Writing- Original Draft Preparation; Denys Vatlitsov: Conceptualization, Methodology, Validation, Investigation, Software, Writing - Original Draft Preparation; Nadiia Dovgopola: Writing - Reviewing and Editing; Alla Tkachenko: Resources; Mariya Klunnyk: Writing – Reviewing and Editing
